# Indigenous versus Lessepsian Hosts: Nervous Necrosis Virus (NNV) in Eastern Mediterranean Sea Fish

**DOI:** 10.3390/v12040430

**Published:** 2020-04-10

**Authors:** Yael Lampert, Ran Berzak, Nadav Davidovich, Arik Diamant, Nir Stern, Aviad P. Scheinin, Dan Tchernov, Danny Morick

**Affiliations:** 1 Department of Marine Biology, Leon H. Charney School of Marine Sciences, University of Haifa, Haifa 3498838, Israel; yael.lampert.kelman@gmail.com (Y.L.); ranberzak@gmail.com (R.B.); arik.diamant@gmail.com (A.D.); shani.aviad@gmail.com (A.P.S.); dtchernov@univ.haifa.ac.il (D.T.); 2 Morris Kahn Marine Research Station, University of Haifa, Haifa 3498838, Israel; 3 Israeli Veterinary Services, Bet Dagan 5025001, Israel; Nadavd@moag.gov.il; 4 National Institute of Oceanography, Israel Oceanographic and Limnological Research, Haifa 3108001, Israel; nirstern@ocean.org.il

**Keywords:** viral diseases, fish, nervous necrosis virus (NNV), Lessepsian species, indigenous species, Mediterranean Sea

## Abstract

Viruses are among the most abundant and diverse biological components in the marine environment. In finfish, viruses are key drivers of host diversity and population dynamics, and therefore, their effect on the marine environment is far-reaching. Viral encephalopathy and retinopathy (VER) is a disease caused by the marine nervous necrosis virus (NNV), which is recognized as one of the main infectious threats for marine aquaculture worldwide. For over 140 years, the Suez Canal has acted as a conduit for the invasion of Red Sea marine species into the Mediterranean Sea. In 2016–2017, we evaluated the prevalence of NNV in two indigenous Mediterranean species, the round sardinella (*Sardinella aurita*) and the white steenbras (*Lithognathus mormyrus*) versus two Lessepsian species, the Randall’s threadfin bream (*Nemipterus randalli*) and the Lessepsian lizardfish (*Saurida lessepsianus*). A molecular method was used to detect NNV in all four fish species tested. In *N. randalli*, a relatively newly established invasive species in the Mediterranean Sea, the prevalence was significantly higher than in both indigenous species. In *S. lessepsianus*, prevalence varied considerably between years. While the factors that influence the effective establishment of invasive species are poorly understood, we suggest that the susceptibility of a given invasive fish species to locally acquired viral pathogens such as NVV may be important, in terms of both its successful establishment in its newly adopted environment and its role as a reservoir ‘host’ in the new area.

## 1. Introduction

Marine organisms typically host diverse communities of viruses, bacteria, archaea, and fungi [1,2,3]. Marine viruses are key drivers of host diversity, population dynamics, and biogeochemical cycles, and, therefore, their effect on the marine environment is extensive [2,4]. Viral encephalopathy and retinopathy (VER) is a disease caused by nervous necrosis virus (NNV), an RNA virus belonging to the genus *Betanodavirus* (Nodaviridae). NNV is globally distributed and has been detected in 177 susceptible marine species, while epizootic outbreaks have been reported in 62 of them [5]. The disease has caused ecological and economical losses worldwide in a variety of reared marine fish species, but also in freshwater species worldwide [5,6,7,8]. The opening of the Suez Canal in 1869 is considered an important event in many respects, especially from the biogeographical perspective. In particular, it facilitated marine faunal invasions from the Red Sea, inducing dramatic changes in the eastern Mediterranean coastal ecosystems [7]. This influx of invasive species into the Mediterranean Sea, termed “Lessepsian migration”, has hitherto been well documented [8,9]. Among all taxa, due to their ecological and commercial importance, invasive fish species have been the most extensively studied, considering their well-elucidated and documented taxonomy [10,11]. The success of a nonindigenous species in a new habitat is often explained by the “enemy release hypothesis”. According to this theory, during the process of biological invasion, nonindigenous species are released from the effects of the natural enemies (for example competitors, predators, and pathogens) which control their population in their native region. The idea that exotic species may leave their natural enemies behind has a long history [12,13], and it has been suggested that the release from pathogens may have great relevance to this phenomenon [14,15,16,17]. Pathogens can directly affect their hosts by reducing growth, curbing reproduction rates, and diminishing survival, or indirectly, by influencing behaviors and competition [18]. Thus, like most natural enemies, pathogens can regulate their host populations in a density-dependent way [19,20]. 

The genome of NNV consists of two segments, RNA1 (3.1 Kb) and RNA2 (1.4 Kb), which code for the RNA-dependent RNA polymerase and the coat protein. Based on a phylogenetic analysis of the T4 variable region within the RNA2 segment, *Betanodaviruses* have been clustered into four genotypes, currently accepted by the International Committee on Taxonomy of Viruses (ICTV) as official species of this genus: striped jack nervous necrosis virus (SJNNV), tiger puffer nervous necrosis virus (TPNNV), barfin flounder nervous necrosis virus (BFNNV), and red-spotted grouper nervous necrosis virus (RGNNV) [5].

In our previous study [21], NNV was found in wild and cultured fish and crustaceans from the eastern part of the Mediterranean Sea. While NNV prevalence levels varied among different fish species, initial findings indicated a relatively high prevalence in Lessepsian fish species compared to Mediterranean indigenous species. Following this preliminary survey, the prevalence of NNV has been studied over an additional one year period and compared to our previous results [21]. Four fish species were studied: two indigenous species, the round sardinella (*Sardinella aurita*) and the white steenbras (*Lithognathus mormyrus*), and two Lessepsian species, Randall’s bream (*Nemipterus randalli*) and lizardfish (*Saurida lessepsianus*). These four species were selected due to their ecological and economic importance as major species in the eastern Mediterranean fisheries. In addition, NNV detected in various species was genetically characterized.

## 2. Materials and Methods

### 2.1. Fish and Tissue Sampling

In October–December 2017, a total of 109 specimens of four wild fish species: *L. mormyrus* (n = 20), *S. aurita* (n = 28), *N. randalli* (n = 31), and *S. lessepsianus* (n = 30) were caught and brought on ice to the laboratory. All specimens were caught along the Israeli Mediterranean shoreline by bottom trawling during the annual national monitoring project conducted in November 2017 by the Israel Oceanographic and Limnological Research Institute (IOLR) and during the surveys conducted between October to December 2017 by the Fisheries and Aquaculture Department of the Israel Ministry of Agriculture. Weight and Total Length measurements were taken, and visual inspections were carried out. All specimens were aseptically dissected and brain tissue samples were collected according to fish necropsy protocol [22]. All samples were kept frozen at −80 °C until further analysis.

### 2.2. RNA extraction and cDNA synthesis by RT-PCR 

Total RNA from brain tissue was extracted by using the EZ-RNA total RNA isolation kit (Biological Industries) according to the manufacturer’s instructions. The RNA concentration and quality were estimated using NanoDrop One (Thermo Scientific), and the extracted RNA was stored at −80 °C until use. Real-time Reverse Transcription PCR (RT-PCR) was performed to generate cDNA of total RNA using the GoScript^TM^ Reverse Transcription System (Promega, WI, USA) with random primers according to the manufacturer’s instructions.

### 2.3. RNA1 and RNA2 PCR amplification

For genotyping the NNV strains, all samples were subjected to PCR targeting both genomic segments using primers previously described by [23,24] targeting a 630 nt fragment of the RNA1 segment FOR521: 5′-ACG TGG ACA TGC ATG AGT TG-3′ and VNNV6: 5′-ACC GGC GAA CAG TAT CTG AC-3’, and primers targeting a 605 nt fragment of the RNA2 segment VNNV1: 5′-ACA CTG GAG TTT GAA ATT CA-3′ and VNNV2: 5′-GTC TTG TTGAAG TTG TCC CA-3′. PCRs were performed in reaction tubes preloaded with 2 μL of cDNA template, 0.5 μL (10 mM) of each primer, 9.5 μL of ultra-pure PCR water, and 12.5 μL GoTaq Green Master mix (Promega) on a SimpliAmp Thermal Cycler (Applied Biosystems). The PCR thermal profile for RNA1 was one cycle of 95 °C for 4 min, 35 cycles of 95 °C for 30 s, 57 °C for 30 s, 72 °C for 30 s, and a final extension of 72 °C for 10 min. The PCR reaction thermal profile for RNA2 was one cycle of 95 °C for 4 min, 35 cycles of 95 °C for 30 s, 51 °C for 30 s, 72 °C for 30 s, and a final extension of 72 °C for 10 min. 

### 2.4. Sequencing and Phylogenetic Analysis

PCR amplicons were purified by ExoSAP-IT (Affymetrix), followed by Sanger sequencing at the Macrogen Europe Laboratory (Macrogen Inc., The Netherlands/South Korea). Sequences were aligned using the MEGA7 software [25] and compared by BLAST to representative sequences available in GenBank. Sequences of NNV isolates from wild and farmed fish from the Mediterranean area, including two from the Israeli Mediterranean and Red Sea (only RNA2 sequences were available), were included in the phylogenetic analysis (Table 1). Genetic characterization of NNV positive isolates, based on RNA1 and RNA2, were inferred from maximum likelihood (ML) trees performed with the PhyML v.3.0 program [26] by applying the K80 + I model of nucleotide substitution for RNA1 and RNA2, based on the Smart Model Selection [27] available in the PhyML program. The robustness of the nodes on the phylogeny was assessed by 1000 bootstrap replicates using the ML substitution model defined above. Phylogenetic trees were visualized with the FigTree v1.4.3 software (http://tree.bio.ed.ac.uk/software/figtree/). 

### 2.5. Statistical Analysis

The statistical significance of the prevalence of NNV between various fish species was determined by the Chi-Square test of independence using IBM SPSS Statistics for Windows, version 20.0 (IBM Corp., Armonk, NY, USA). For all tests, a *p*-value < 0.05 was considered significant.

## 3. Results

**Figure 1:**
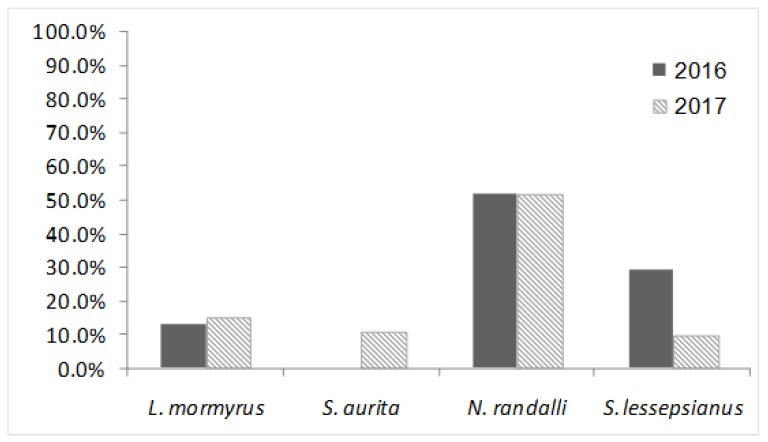
Percentage of NNV positive in the sampled wild fish species. For each species, the results in 2016 and 2017 are presented. No significant difference was found between the sampled years per each species (Chi-Square test of independence, *p* > 0.05).

**Figure 2:**
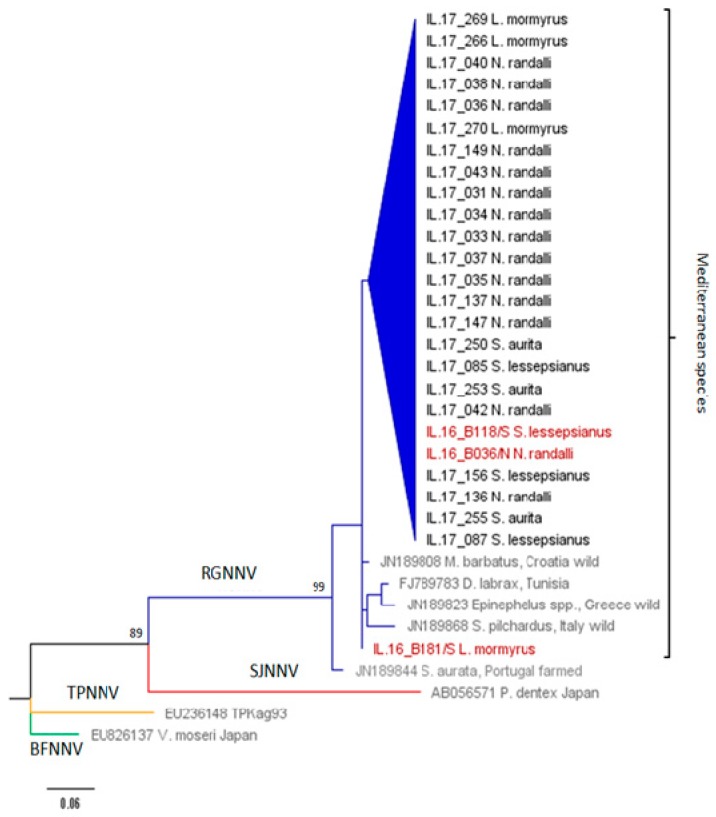
RNA1 phylogenetic tree. Maximum likelihood (ML) phylogenetic tree of the RNA1 partial sequences. The sequence name includes the identification number and host species. Positive samples of this study (begins with IL) are in black, reference sequences from GenBank are in grey, and representative sequences from 2016 [21] are in red. NNV genotype subdivision is displayed on the branches (blue: RGNNV; green: BFNNV; yellow: TPNNV; red: SJNNV).Vertical brace designate subclustering of samples from the Mediterranean Sea. The numbers at the branches nodes represent bootstrap values (only values ≥ 70% are reported). The scale bar represents 0.06 nucleotide substitution per site.

**Figure 3:**
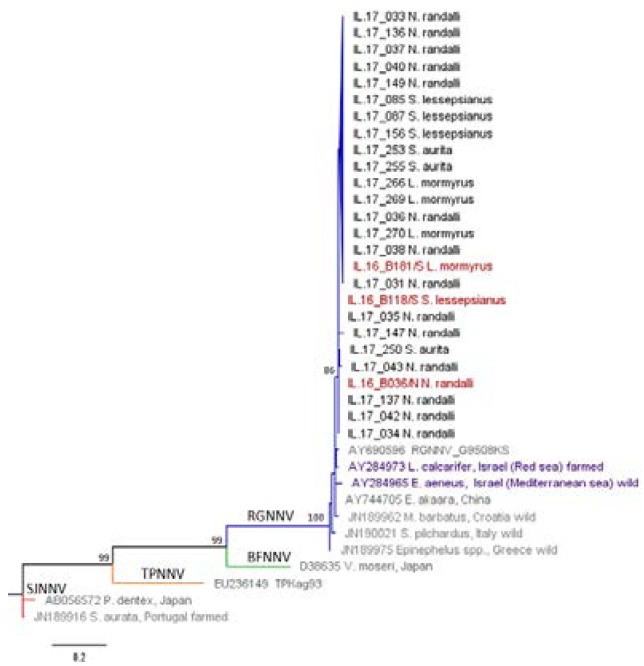
RNA2 phylogenetic tree. Maximum likelihood (ML) phylogenetic tree of the RNA2 partial sequences. The sequence name includes the identification number and host species. Positive samples of this study (begins with IL) are in black, reference sequences from GenBank are in grey or purple for isolates from Israel, and representative sequences from 2016 [21] are in red. NNV genotype subdivision is displayed on the branches (blue: RGNNV; green: BFNNV; yellow: TPNNV; red: SJNNV). The numbers at the branches nodes represent bootstrap values (only values ≥ 70% are reported). The scale bar represents 0.2 nucleotide substitution per site.

All 109 collected wild fish were individually tested for NNV by PCR amplification of both genomic RNA1 and RNA2 segments. Based on the sequencing of PCR amplicons, the overall prevalence of NNV was 22.94%. NNV was present in all four examined wild fish species (Table 2). No significant difference (*p* > 0.05) in total prevalence of NNV was found in the same sampled species when comparing the two consecutive years 2016 [21] and 2017 (Table 2, Figure 1). The two-year prevalence of NNV in *S. lessepsianus*, when comparing to the indigenous species, was significantly higher (χ^2^ = 4.5, *p* < 0.05), whereas in the case of the two-year prevalence of NNV in *N. randalli* as compared to the indigenous species, the higher values were highly significant (χ^2^ = 37.6, *p* < 0.001). Hence, the prevalence of NNV in the Lessepsian species was significantly higher than in the indigenous species in both consecutive years together (χ^2^ = 21.9, *p* < 0.001). However, the main prevalence values for this finding were dominated by *N. randalli*, in which the prevalence of NNV was significantly higher than in the other three species in 2017 alone (χ^2^ = 20.15, *p* < 0.001), as well as in the combined two-year data (χ^2^ = 36.2, *p* < 0.001). The RNA1 and RNA2 phylogenetic analysis of NNV revealed that all samples were clustered with the RGNNV genotype (bootstrap values ≥ 70%) and no reassortants were found (Figure 2 and Figure 3). NNV sequences were deposited in GenBank, with the following accession numbers: 2016—MH970524, MH970531, MH970532, MH970555, MH970563, and MH970564. NNV sequences 2017—MT281320-MT281365.

## 4. Discussion and Conclusions

The prevalence of NNV in 109 individual fish belonging to two indigenous and two Lessepsian species from the eastern Mediterranean Sea was determined. The mean prevalence values of NNV were higher than those found in wild fish populations in previous studies elsewhere in the Atlanto-Mediterranean region, where a *Betanodavirus* prevalence of 11.9% was found in the Adriatic Sea [28] and 19.6% at the Gulf of Cadiz in the Atlantic Ocean, close to the Gibraltar Strait [29]. However, in wild fish sampled adjacent to fish farms in Tunisia, a much higher average prevalence, i.e., 62%, of NNV was detected only [30]. In the case of the described study, it is important to note that the extremely high prevalence detected may be due to the sampling strategy used (sea cages). The present study revealed a significantly high level of NNV in one of the Lessepsian species, *N. randalli*, while the other Lessepsian species, *S. lessepsianus*, indicated a relatively lower prevalence (mean of 20.6% for both years; see Table 2). Both had higher values than the two indigenous species. In a comparable study of the parasites of the Lessepsian bluespotted cornetfish (*Fistularia commersonii*), it was revealed that the parasite assemblage of *F. commersonii*, after migration to the Mediterranean Sea, was reduced in its natural specialist parasites. At the same time, native generalist parasite species were acquired in the new environment, in agreement with the enemy release hypothesis [20]. In the current study, only the prevalence of one pathogen, NNV, was investigated; other natural pathogens of these Lessepsian species were not surveyed. *N. randalli* was first reported in the Mediterranean Sea in 2005 [31], and since then, it has become one of the dominant fish species in the Israeli ichthyofauna [9]. The establishment of invasive species has been shown, among other things, to induce shifts in local biodiversity, alterations of ecosystem processes, changes in food webs, and the introduction of new pathogens [32]. As for the causes for the successful establishment of *N. randalli*, these are not yet understood. Some possible explanations for the establishment of invasive species have been suggested, for example, favorable temperature, foraging activities, and partitioning of biological niche [9,33,34]. Our results suggest that perhaps also an additional factor should be taken into consideration: the sensitivity level of species to pathogens. The pathogenicity of NNV depends on host susceptibility, determined by the RNA2, as well as environmental conditions that are regulated by the RNA1 [35]. Molecules presented on the capsid protein, encoded by RNA2, interact with receptors on the surfaces of target cells and facilitate the entry of the virus [36,37]. After viral entry, the rate of its replication depends in part on temperature, which influences the RNA-dependent RNA polymerase, encoded by RNA1. The host’s short interference RNA, antagonized by its B2 protein, is capable of limiting the accumulation of the virus [38]. Accordingly, it is possible that NNV has a good ability to infect *N. randalli*, but a low ability to produce lethal disease due to different host immune mechanisms [36]. Possibly, the different temperature regimes between the Red Sea and the Mediterranean may influence the rapid replication of NNV in *N. randalli* after invasion. Unfortunately, there are no data on prevalence levels of NNV in *N. randalli* in the Red Sea (nor in any Red Sea fish species). It is well known that the RGNNV genotype, which affects tropical and temperate fish species, is the most widely distributed NNV-genotype, and has a high recorded number of susceptible species [5]. Moreover, RGNNV genotype is widely distributed in both wild and farmed fish species in the Mediterranean Sea and along the coasts of Asia and Australia [5]. The temperature regimes of the Mediterranean Sea [37,38] and the Red Sea [39,40] have been discussed by different authors. It is difficult to compare the two, since measurements showed that water temperatures vary between geographical locations within both seas and are influenced by seasonality. The minimal temperature measured in the Mediterranean Sea was 17 °C [37] in comparison to 22 °C in the Red Sea [39]. However, the maximal temperature measured in Mediterranean Sea was 30 °C [37] in comparison to 34 °C in the southern part of the Red Sea [39]. In this study, fish were collected between October and December 2017; seawater temperatures during this period ranged between 27.1–28.6 °C (historical time series database of IOLR—https://isramar.ocean.org.il/isramar_data/TimeSeries.aspx). In an experimental infection conducted on European seabass (*Dicentrarchus labrax*), it was shown that RGNNV would be more virulent at 25–30 °C than at 20 °C [35]. There is agreement between the seawater temperatures in our study (27.1–28.6 °C) and those described for *D. labrax*. Further studies should determine whether the high prevalence of this virus in the Lessepsian population is due to high resistance of the host, to temperature-related factors, or to other unknown factors that contributed to its successful establishment in the Mediterranean Sea in a relatively short time. We also suggest that further studies should include isolation of the viral agent in order to evaluate whole-genome sequencing (WGS) and provide a wider molecular insight of wild-NNV-isolates. Moreover, the histopathology of these wild fish species in future studies will enable us to better understand whether the histopathological manifestation in wild species differs from our knowledge of common mariculture species. This study warrants further investigations to try isolating the virus to determine whether it is viable, and to perform a histopathological analysis in order to verify whether it causes damage to the nervous tissues of these two Lessepsian species.

The phylogenetic analysis of NNV across the four wild fish species from the Mediterranean Levantine Basin showed that all belonged to the RGNNV genotype. These results are in agreement with previous studies that reported the presence of RGNNV genotype only and revealed no reassortant genotypes in wild species [29,41,42,43,44]. 

Another viral disease that was detected in both the Mediterranean and Red Seas is *Lymphocystis* Disease Virus (LCDV), which belongs to the family *Iridoviridae*. Iridoviruses possess a 20-sided icosahedron with a DNA core, and the particle is relatively large, measuring from 120–300 nm in diameter. The *Lymphocystis* virus causes a self-limiting disease in young fish [45,46]. An outbreak of *Lymphocystis* disease was described in 2008, affecting cultured gilthead seabream (*Sparus aurata*) in Eilat land-based nursery tanks (Red Sea) [45]. Later, in 2013, *Lymphocystis* disease was reported in reared gilthead seabream (*Sparus aurata*) along the Tunisian coast [46]. Further studies investigating NNV in wild Lessepsian and indigenous species from both seas are necessary.

This study reported the high prevalence of NNV in the Lessepsian species *N. randalli*, and suggests that viral resistance should be considered as an additional mechanism that may favor the establishment of invasive species in their new environment. While the factors that influence effective establishment are still poorly understood [15,16], it is clear that multiple opposing factors interact and work to facilitate or impede the successful establishment of invasive species. We suggest that the resistance of a given invasive fish species to locally acquired pathogens (such as NNV in this case) can contribute to the success of the host in its newly adopted environment. This suggestion is in agreement with the concept that invasive fish species rid themselves of some of the original (natural) parasitic load during invasion, as previously suggested [14,17]. Can this mechanism work in the opposite direction and be detrimental to the establishment of virus-susceptive invasive species in a new area? Clearly, further studies are needed to better understand the underlying causes for the successful establishment of invasive species in their new environment, as well as to understand the reciprocal pathogen transmission dynamics between indigenous and invasive species.

## Figures and Tables

**Table 1:** List of NNV isolates used in the phylogenetic analysis. Host fish species or isolate number, Country and origin, wild or farmed, viral strain, and accession numbers in GenBank.

Host Species/Isolate	Origin	NNV Strain	RNA1	RNA2
*Epinephelus akaara*	China	RGNNV	-	AY744705
G9508KS	n.a	RGNNV	-	AY690596
*Dicentrarchus labrax*	Tunisia	RGNNV	FJ789783	-
*Sardina pilchardus*	Italy, wild	RGNNV	JN189868	JN190021
*Epinephelus spp.*	Greece, wild	RGNNV	JN189823	JN189975
*Mullus barbatus*	Croatia, wild	RGNNV	JN189808	JN189962
*Epinephelus aeneus*	Israel (Med. Sea ^b^), wild	RGNNV	-	AY284965
*Lates calcarifer*	Israel (Red Sea), farmed	RGNNV	-	AY284973
*Sparus aurata*	Portugal, farmed	RG/SJ ^a^	JN189844	JN189916
*Pseudocaranx dentex*	Japan	SJNNV	AB056571	AB056572
*Verasper moseri*	Japan	BFNNV	EU826137	D38635
TPKag93	Japan	TPNNV	EU236148	EU236149

^a^ Genotyping of RNA1/RNA2 of NNV. ^b^ Mediterranean Sea. n.a.: not available

**Table 2:** Results of 2016 and 2017 surveys for NNV prevalence in wild fish species from the Mediterranean Sea. Results based on PCR targeting RNA1 and RNA2 segments. No significant difference was found between the same species in the consecutive years (Chi-Square test of independence, *p* > 0.05).

	2016			2017			Total		
Fish Species	n	Positive	% Prevalence	n	Positive	% Prevalence	n	Positive	% Prevalence
*Lithognathus mormyrus*	30	4	13.33	20	3	15	50	7	14
*Sardinella aurita*	30	0	0	28	3	10.71	58	3	5.17
*Nemipterus randalli* *	29	15	51.72	31	16	51.61	60	31	51.67
*Saurida lessepsianus* *	38	11	28.95	30	3	10	68	14	20.59
Total	127	30	23.62	109	25	22.94	236	55	23.31

* indicates an invasive Red-Sea Indo-pacific species.
